# A phase I clinical trial of adoptive transfer of folate receptor-alpha redirected autologous T cells for recurrent ovarian cancer

**DOI:** 10.1186/1479-5876-10-157

**Published:** 2012-08-03

**Authors:** Lana E Kandalaft, Daniel J Powell, George Coukos

**Affiliations:** 1 Ovarian Cancer Research Center, Perelman School of Medicine at University of Pennsylvania, Philadelphia, PA, USA

## Abstract

**Purpose:**

In spite of increased rates of complete response to initial chemotherapy, most patients with advanced ovarian cancer relapse and succumb to progressive disease.

**Rationale:**

Genetically reprogrammed, patient-derived chimeric antigen receptor (CAR)-T lymphocytes with the ability to recognize predefined surface antigens with high specificity in a non-MHC restricted manner have shown increasing anti-tumor efficacy in preclinical and clinical studies. Folate receptor-α (FRα) is an ovarian cancer-specific tumor target; however, it is expressed at low levels in certain organs with risk for toxicity.

**Design:**

Here we propose a phase I study testing the feasibility, safety and preliminary activity of FRα-redirected CAR-T cells bearing the CD137 (4-1BB) costimulatory domain, administered after lymphodepletion for the treatment of recurrent ovarian cancer. A novel trial design is proposed that maximizes safety features.

**Innovation:**

This design involves an initial accelerated dose escalation phase of FR-α CAR-T cells followed by a standard 3 + 3 escalation phase. A split-dose approach is proposed to mitigate acute adverse events. Furthermore, infusion of bulk untransduced autologous peripheral blood lymphocytes (PBL) is proposed two days after CAR-T cell infusion at the lower dose levels of CAR-T cells, to suppress excessive expansion of CAR-T cells in vivo and mitigate toxicity.

## Background and rationale

### Adoptive T cell therapy in ovarian cancer

Ovarian cancer is the fifth most common cancer in women, affecting one of every 55 women. There are about 21,650 new cases annually in the United States, with 15,520 deaths estimated in 2008 [1] making ovarian cancer the most common cause of death from gynecologic malignancy. While there have been improvements in the treatment of epithelial ovarian cancer, most patients present with Stage III or IV disease, which has a 5-year survival rate of less than 25% [2-4]. New approaches are needed to improve the outcome of these women [5].

Adoptive immunotherapy is one of the most robust forms of immunotherapy for the treatment of established tumors [6]. Early cell transfer trials in ovarian cancer have been promising: In one such trial, administration of autologous tumor-infiltrating lymphocytes (TILs) to ovarian cancer patients after surgical resection and cisplatin chemotherapy resulted in prolonged disease-free survival and increased the 3-year survival rate, supporting the notion that T cell transfer can actively inhibit ovarian tumor growth [7]. In another study, administration of TILs (alone or in combination with chemotherapy) was shown to induce objective cancer regressions [8]. Although adoptive immunotherapy has much promise, several problems remain to be solved (reviewed in [5,6,9]).

One major obstacle facing the field of cancer immunotherapy is the daunting task of breaking tolerance to self-antigens. This can be difficult or impossible if the T cell receptor (TCR) repertoire has been deleted or rendered non-functional by various post-thymic tolerance mechanisms [10,11]. Several strategies for identifying therapeutically effective T cell clones and expressing the operative heterodimeric TCR in patients’ lymphocytes prior to autologous transplant have been tested (reviewed in [12-16]). An alternative strategy to produce genetically engineered T cells is the ‘T-body’ or chimeric antigen receptor (CAR) [17] approach.

CARs are fusion molecules comprising an extracellular binding domain, typically a single-chain variable-fragment antibody (scFv), containing the V_H_ and V_L_ chains joined by a peptide linker of about 15 residues in length [18] and intracellular lymphocyte signaling domains such as CD3ζ, CD28, 4-1BB (CD137), which mediate T cell activation (reviewed in [14]). CARs bypass a common immune evasion mechanism of tumor cells, the downregulation of MHC-I and antigen presentation, and provide unique opportunities to engineer T cells without MHC restriction and with potent costimulatory signals.

### CAR-T cell therapy targeting FRα

A large number of CARs targeting diverse tumors have been developed [14]. Although clinical pilot studies are just beginning, the potential of this form of immunotherapy is becoming increasingly obvious. A trial of 11 patients with neuroblastoma treated with CAR-T cells specific for the GD2 ganglioside showed short-term persistence of CARs with some evidence of antitumor effects [19]. In another report, 19 patients were treated with GD2-specific CAR-T cells, and 3 of 11 achieved remission with long-term persistence of the CARs, which appeared to correlate with clinical outcome [20]. Initial reports have also documented remarkable clinical responses in patients with advanced chronic lymphocytic leukemia (CLL) or lymphoma after therapy with CD19-specific CAR-T cells [21-25]. There has been a single study of adoptive transfer of CAR-T cells in ovarian cancer, with specificity directed against folate receptor-α (FRα) [26]; while this study demonstrated safety, the results were disappointing, with no clinical tumor responses, most likely due to low expression of the transgenic CAR, and poor persistence of the transferred T cells.

For the treatment of ovarian cancer, an appropriate target is the FRα, a membrane protein, which binds folic acid with high affinity and mediates the cellular uptake of this vitamin (and drug conjugates thereof) via receptor-mediated endocytosis [27]. Subsequent analyses have shown elevated FRα expression in approximately 90% of ovarian carcinomas, as well as, numerous other cancers, including endometrial, kidney, lung, mesothelioma, breast, brain, and myeloid leukemia, whereas most normal tissues express low to negligible levels [28]. This could be of clinical significance, particularly given the current development of folate-targeted imaging and therapeutic agents [29]. Low-level FRα expression in normal human tissues is limited to the luminal surface of cells located in the bronchial epithelium, renal tubules and the choroid plexus, intestinal brush-border membranes, type-1 and type-2 pneumocytes of the lung, and placental tissue. These folate receptors remain inaccessible to folate and folate conjugates because of their exclusive localization on the apical surfaces of polarized epithelia, and consequently, in healthy patients that do not have malignant masses or an inflammatory disease, measurable folate conjugate uptake has been limited to the kidneys where folate salvage occurs [30]. Renal toxicity has not been observed in animals or humans treated with folate-chemotherapeutic agent conjugates [31,32], antibodies [33]or directed T cells [26,34], however the acute, transient toxicities associated with administration of anti-folates, such as methotrexate, bear consideration. To date, various folate–drug conjugates have demonstrated safety and therapeutic value in animals [8,35] and in phase-I trials [30]. Promising pilot efficacy data of treating ovarian cancer patients with FRα-directed T cells was provided by a study of adoptive, intraperitoneal (IP) transfer of armed autologous T cells retargeted to FRα using a bispecific monoclonal antibody containing anti-CD3 and anti-FRα MOv18 F(ab’)2 fragments, in combination with IP interleukin-2 (IL-2) [34]. The overall intraperitoneal response rate was 27%, with three complete responses lasting 26, 23 and 18 months, respectively. Adverse events included grade 1–2 fever, nausea, emesis and fatigue, which appeared related to IL-2 administration and not to FRα targeting. In the first reported clinical trial of CAR therapy, administration of T cells transduced with a MOv18 scFv-directed FRα-specific CAR harboring no costimulatory domains resulted in poor persistence of transferred T cells in immunocompetent recipients in vivo [26]. While this study demonstrated safety, the results were disappointing, with no clinically evident tumor responses.

**Figure 1:**
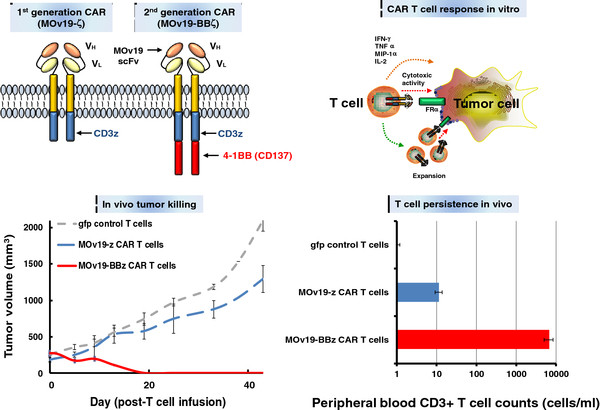
**Schematic summary of preclinical results for FRα-specific CAR T cell therapy.** (*Upper left*) Cartoon of first (MOv19-ζ) or second generation (MOv19-BBζ) FRα-specific CARs bearing CD3ζ alone or in combination with a 4-1BB costimulatory domain, respectively. (*Upper right*) Summary of in vitro CAR effects. FRα-specific CAR T cells produce proinflammatory cytokines, proliferate and kill FRα-expressing tumor cells in vitro. (*Lower left*) Administration of second generation MOv19-BBζ CAR T cells results in eradication of human FRα ovarian cancer cells in immunodeficient mice. (*Lower right*) Tumor regression in vivo was associated with increased human T cell persistence in the blood 3 weeks after infusion.

In the proposed study, we will test the safety and efficacy of lymphodepletion followed by adoptive transfer of autologous T cells transduced with CAR recognizing the FRα, and carrying the CD3ζ domain along with the 4-1BB costimulatory signaling domain to address the issue of persistence of FRα-specific CAR-T cells. This CAR construct was developed by Powell and colleagues and was tested in an animal model [35]. Preclinical results show clearly that the anti-FRα CAR outfitted with intracellular CD3ζ and 4-1BB costimulatory signaling overcomes issues of poor T cell persistence and tumor localization that limited the previous FRα CAR-T cell targeting strategy, to provide potent antitumor activity in vivo [35]. CARs containing the FRα-specific scFv MOv19 coupled to the T cell receptor CD3ζ chain signaling module alone (MOv19-ζ) or in combination with the 4-1BB (CD137) costimulatory motif in tandem (MOv19-BBζ) were directly compared (summarized in Figure 1). Primary human T cells that were stably transduced with recombinant lentivirus to express a first generation MOv19-ζ or second generation costimulated MOv19-BBζ CAR secreted various proinflammatory cytokines and exerted cytotoxic function when cocultured with FRα-positive tumor cells in vitro. However, only transfer of human T cells expressing the costimulated MOv19-BBζ CAR mediated tumor regression in immunodeficient mice bearing large, established FRα-positive human ovarian cancers, while MOv19-ζ CAR-T cells were ineffective, recapitulating previous clinical results with FRα CAR T cells lacking costimulatory signals. MOv19-BBζ CAR-T cell infusion mediated tumor regression in models of metastatic intraperitoneal, subcutaneous, and lung-involved human ovarian cancer. Importantly, tumor response was associated with the selective survival and tumor localization of human T cells in vivo, and was only observed in mice receiving costimulated MOv19-BBζ CAR-T cells. These compelling data warrant the translation to the clinic, and led us to design the following phase I protocol.

### Trial design

This is a phase I, single-arm, single-center, dose-escalation study to establish the safety and proof of concept of autologous FRα-redirected T cells administered intravenously in subjects with recurrent stage II to IV FRα-positive epithelial ovarian carcinoma. Subjects will be 18 years or older diagnosed with advanced ovarian cancer who have failed two or more prior chemotherapy regimens, with ECOG ≤2 performance status, and >3 month expected survival.

This design involves an initial accelerated dose-escalation phase followed by a standard 3 + 3 escalation phase. In the initial accelerated phase, cohorts of one subject will be treated per dose level. The accelerated phase will end when the first instance of moderate toxicity (MT) attributed to T cell reactivity at any dose level is observed. The cohort at the current dose level will be expanded and the standard 3 + 3 phase will ensue for all subsequent cohorts.

#### The accelerated escalation phase

Consists of one cohort, divided into 3 subcohorts of 1 subject per dose level (Cohorts 1A, 1B, and 1 C). CAR-T cells will be given to lymphodepleted subjects by a single intravenous infusion on Day 0. Three dose levels will be tested, starting at 3 million CAR-T cells; followed by 10 million CAR-T cells; with the final cohort receiving 30 million CAR-T cells. All doses are +/− 20%, and at least 10% of T cells in the preparation must be CAR-positive to meet release criteria. In a lymphopenic host, adoptively transferred T cells (including CAR-T cells) are expected to expand exponentially until homeostatic restoration of the T cell counts. To contain the exponential expansion of CAR-T cells and maximize safety, one billion autologous untransduced peripheral blood lymphocytes (PBL) will be administered intravenously on Day 2 to all subjects in the accelerated escalation phase (cohort 1A, 1B, and 1 C). Additional safety benefits will be derived by the significantly shortened lymphopenia under this design.

#### The standard escalation phase

**Figure 2:**
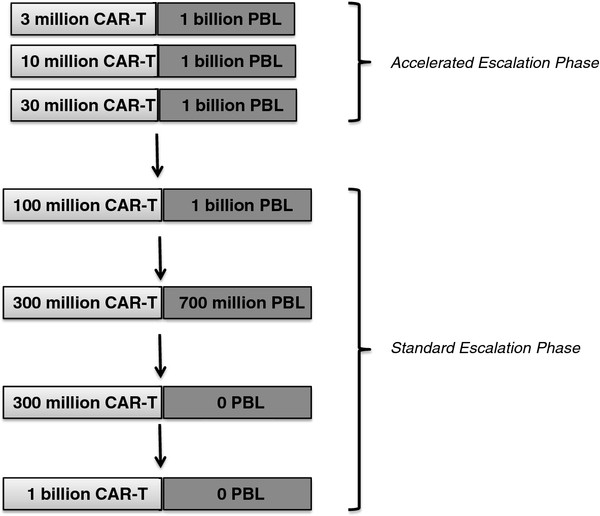
Clinical trial design: Dose escalation schema.

Consists of 4 cohorts, each comprising 3 patients per dose level. To maximize safety, CAR-T cells will be dosed using a “split-dose” approach to the intravenous infusion: 10% on Day 0, 30% on Day 1 and 60% on Day 2. Four dose levels will be given, from 100 million to 1 billion CAR-T cells. Again, to prevent the population of CAR-T cells from expanding excessively, subjects will be administered untransduced PBL in a “decrescendo” dose scheme: cohort 2 will receive 100 million CAR-T cells followed by 1 billion PBL; cohort 3 will receive 300 million CAR-T cells followed by 700 million PBL; cohorts 4 and 5 will recieve 300 million and 1 billion CAR-T cells, respectively, without any PBL (Figure 2).

#### Regimen

At entry, subjects will be screened and their eligibility will be determined. Those who meet all eligibility criteria will undergo apheresis within 4–6 weeks prior to the Day 0 visit, in order to obtain peripheral blood mononuclear cells (PBMCs) for CAR-T cell manufacturing. PBL will be enriched by a process of elutriation [36]; transduced with anti-FR-α scFv CAR; expanded in vitro; and then frozen for future administration. Subjects will be allowed to undergo standard of care therapy at the discretion of the investigator while CAR-T cells are being manufactured, if clinically indicated due to rapid disease progression.

Subjects will be enrolled serially, with a minimum of four weeks elapsing between dosing the last subject of one dose level to dosing the first subject at the next dose level. In each cohort, a minimum of three weeks must elapse (for evaluation of toxicities) after each subject starts treatment prior to the next subcohort/subject.

**Figure 3:**
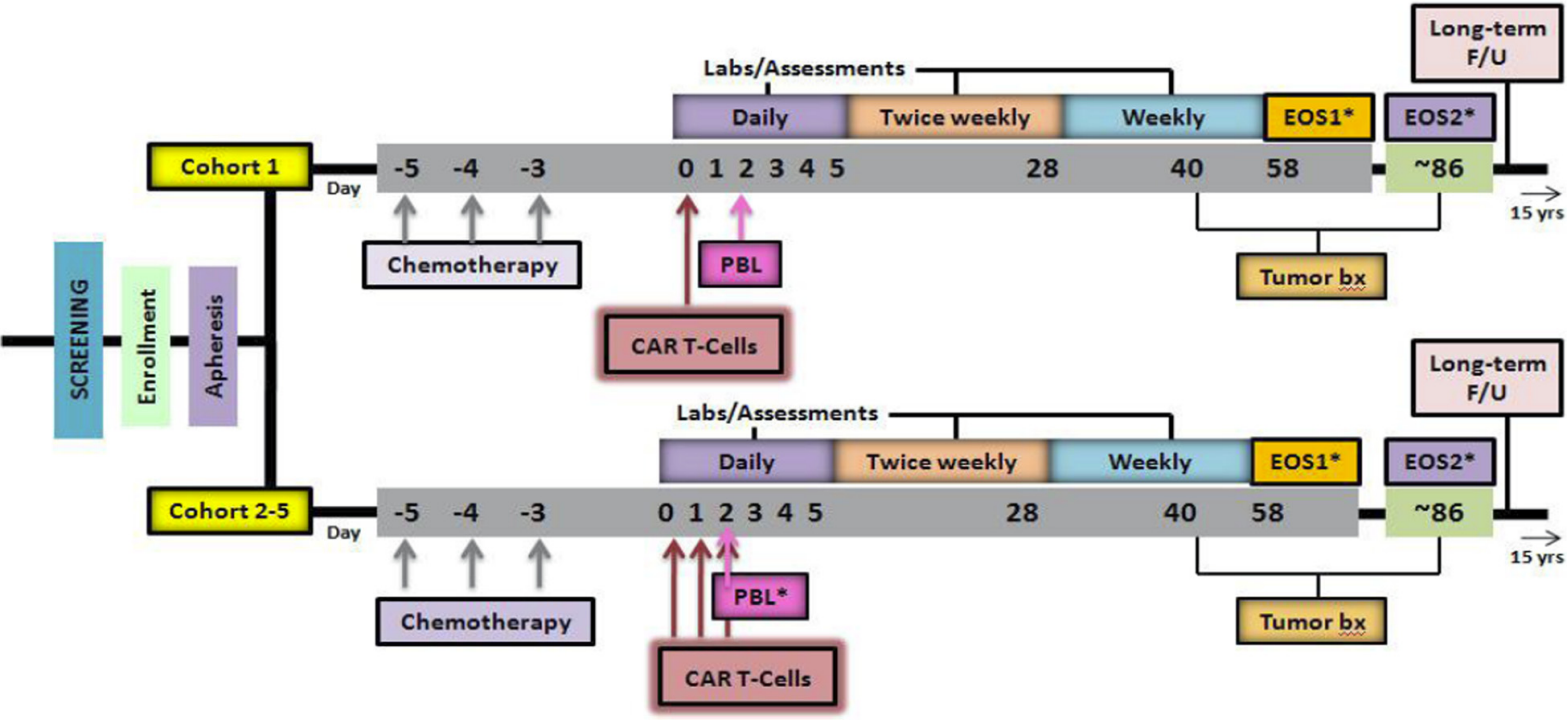
Clinical trial regimen: Schedule of events.

Subjects will receive a single course of outpatient lymphodepleting chemotherapy with intravenous cyclophosphamide (300 mg/m [2]/d for 3 consecutive days) and intravenous fludarabine (30 mg/m [2]/d for 3 consecutive days) prior to T cell administration. Subjects in cohort 1 will receive 3–30 million CAR-T cells dosed intravenously 1–3 days after lymphodepletion on Day 0. Subjects in cohort 2–5 will receive 100 million to 1 billion CAR-T cells 1–3 days after lymphodepletion (on Day 0, 1 and 2), using a “split-dose” approach. Subjects in cohort 1–3 only will receive untransduced autologous PBL intravenously on Day 2. (Figure 3)

Subjects will be followed daily for the first 5 days (starting at Day 0), twice weekly until week 4 (~ 28 Days) and weekly until end of study (EOS). After EOS, the interventional portion of the protocol ends and long-term follow-up (LTFU) begins. LTFU occurs semiannually for up to five years post-infusion and then annually thereafter for up to 15 years after EOS or early withdrawal.

Subjects will have an optional CT or ultrasound-guided tumor biopsy at screening, if they have detectable disease that can be safely accessed, or a needle aspiration of malignant effusion, to determine if tumor expresses FRα. Alternatively, archived tumor tissue will be tested. All subjects will have a mandatory CT or ultrasound-guided tumor biopsy at EOS, if they have detectable disease that can be safely accessed, or a needle aspiration of malignant effusion. Subjects will have end of study 1 visit (EOS1) on ~ Day 58. However, subjects with no evidence of response by conventional RECIST (response evaluation criteria in solid tumors) will be offered to undergo another CT or MRI 4–8 weeks post Day 58 to further evaluate tumor burden applying immune-related response criteria (irRC) [37] to report as a secondary endpoint.

The trial has been approved by all regulatory boards and it will be conducted in accordance with the protocol.

#### Objectives and endpoints

The primary objective of this study is to determine the safety and feasibility of the therapy with T cells stably transduced with the anti-FRα CAR administered intravenously following transient lymphodepletion with cyclophosphamide/fludarabine. The secondary objectives are to determine anti-tumor response using RECIST and/or irRC; to assess the distribution of immune related (ir)-progression-free survival, overall survival and time to progression; to define a no observable adverse effect level (NOAEL) or an optimal biologic dose (OBD) of FRα CAR-T cells; to determine the effect of CAR-T cells on tumor immunity and FRα expression (for patients with available pre-treatment tumor sample); determine whether circulating CAR-T cells retain antitumor effects in time; and to evaluate the engraftment of CAR-T cells in circulation longitudinally in time, and in tumors at the end of study.

#### Statistical methods

This is a phase-I dose-finding study. The sample size is based on the numbers of patients needed to find the MTD and determine with 95% confidence that the toxicity level at the MTD is less than 33%. Safety of transferring T cells will be evaluated through the identification of a phase-II dose using a 3 + 3 standard dose escalation design. Progression-free and overall survival will be presented graphically using the Kaplan-Meier method. Descriptive statistics will be applied to determine the relative engraftment, persistence in blood (and optionally trafficking to tumor) of the CAR-T cells. Number of CAR-T cells in blood, Human Anti-Chimeric antibody (HACA) levels, serum cytokine levels and IFN-g ELISPOT measurement of whole PBL reactivity to FR-α protein will be displayed graphically as a function of time.

#### Translational studies

a) Persistence, engraftment, phenotype and function of CAR + T cells: Our main translational endpoint is to determine the fate of FRα-directed CAR + T cells in vivo. Persistence of T cells post-transfer is a critical marker of successful engraftment and clinical efficacy [38]. Conveniently, CAR + T cells can be readily identified by flow cytometry using PE conjugated goat anti-mouse IgG F(ab')2 (Jackson ImmunoResearch) as well as detected by DNA quantitative (q)PCR for vector copy number in PBMC, an exquisitely sensitive method to test for persistence of CAR + T cells. CAR + T cells will be quantified in peripheral blood longitudinally (2 h post each infusion, daily on Days 4–9, then weekly x 3, every 4 weeks until Month 6, and then every 3 months for 2 years. Relevant to T cell persistence, we will quantify endogenous IL-7 and IL-15 serum cytokines, which have been implicated in the expansion and persistence of transferred T cells in the lymphodepleted host [39], along with a comprehensive cytokine panel using Luminex. The levels of endogenous IL-7 following our outpatient CY/FLU regimen have never been studied and results could guide decisions related to adding adjuvant cytokines (see below). CAR + T cells will also be phenotyped for IL-7 receptor (CD127) and IL-15 receptor alpha expression.

A second important question relates to the phenotype and function of CAR + T cells. Phenotypic analysis of CAR + T cells will include detailed interrogation for memory cell markers (CCR7, CD62L, CD45RA, CD27, CD28, Fas, etc.) vs. effector cell markers (CD45R0, CCR6, CD25, CD38, HLA-DR, GITR, PD-1, etc.), and among these we will quantify CAR + T cells expressing inhibitory receptors suggesting exhausted phenotype (PD-1, LAG-3, TIM-3 etc.). The presence of memory T cells is a strong predictor of long-term persistence and efficacy in the human, and in mouse models of adoptive therapy it has been dramatically enhanced by IL-7 [40]. Phenotypic analysis will be paired with functional analysis; whole PBL or enriched CAR + T cells will be coincubated with ovarian cancer cells expressing FRα, or stimulated with PHA-ionomycin, FRα protein or CD3/CD28 beads as control, followed by interrogation of intracellular cytokines (INF-γ, TNF-α, MIP-1α, IL-2, IL-17, IL-4, TGF-β, IL-10), granzymes, CD137 and CD107a. These will provide a detailed longitudinal characterization of in vivo T cell polarization and function post-transfer. Based on mouse data, we expect these cells to maintain a Th1 polarization, but it is possible that with time they exhibit an exhausted phenotype and function (PD-1+, unable to proliferate and with reduced IL-2 or TNF-α upregulation). These findings will guide future clinical development. Lastly, the presence of CAR + T cells will be quantified in tumor biopsies by DNA qPCR and correlated with tumor FRα protein expression at baseline and end of study.

b) CAR immunogenicity: We will assess the development of host immune responses to the CAR T cells by HAMA, HACA and VSV-G ELISA, and correlate with engraftment of CAR + T cells. These results will guide the need for future development of a CAR bearing a human anti- FRα scFv.

### Innovation

This trial introduces several innovations: First, although, a previous CAR against FRα has been tested, this CAR introduces the potent costimulatory domain of 4-1BB. Second, the previous construct utilized a retrovirus, while this CAR is introduced via a lentivirus. Finally, unlike the previous trial, this trial will use cytotoxic chemotherapy to induce transient lymphodepletion. The proposed therapeutic approach is novel and has not been previously tested. In addition to these technological innovations that take advantage of advances made over the past 10 years [22,35,41], this trial is introducing a novel adoptive T cell therapy design that tries to maximize safety and minimize untoward acute effects, as well as, adverse events from uncontrolled long-term expansion of CAR-T cells that are infused following lymphodepletion.

CAR-T cells may induce acute infusion reactions and pathological cytokine responses. On-target acute toxicity has been observed in some trials using CAR-T cells. A patient treated with T cells bearing a Her2/neu-specific CAR:CD28-CD137-ζ died soon after adoptive transfer, probably due to rapid hyperactivation induced by the recognition of normal tissue expressing the same antigen [42]. Moreover, another patient died after preconditioning and adoptive transfer of T cells bearing a CD19-specific CAR:CD28-ζ, although, in this case an underlying infection released due to the cyclophosphamide preconditioning regimen was the most probable cause of this mortality [43]. To mitigate these risks we introduced two safety measures: a) Subjects will receive incrementing doses of CAR-T cells with the first cohort starting at an unusually low dose of 3 million T cells, and we will escalate doses by half log increments. b) CAR-T cells will be dosed using a “split-dose” approach to the intravenous infusion: 10% on Day 0, 30% on Day 1 and 60% on Day 2.

Furthermore, CAR-T cells may initiate autoimmune attack on normal tissues if their target is expressed in vital organs and a critical number of self-reactive T cells is reached. Examples of such self-recognition include myocarditis, nephritis, hepatitis or colitis. Although, in the proposed trial we will start at a very low dose of CAR-T cells, these are expected to expand exponentially in the lymphodepleted host and eventually they could reach a critical mass sufficient for auto-reactivity. Indeed, in the absence of any additional manipulation, the frequency of CAR-T cells may remain unaltered as adoptive T cells repopulate the host. When Porter and colleagues infused a low dose (1.5 × 10 [5] cells per kilogram of body weight) of autologous anti-CD19 CAR-T cells in a patient with CLL, these cells expanded over 3 weeks to a level that was more than 1,000 times as high as the initial engraftment level in vivo. This patient experienced development of tumor lysis syndrome only after CAR-T cells reached a critical threshold several weeks after T cell transfer, and finally complete remission was achieved. These engineered cells persisted at high levels for 6 months in the blood and the bone marrow and continued to express the chimeric antigen receptor.

**Figure 4:**
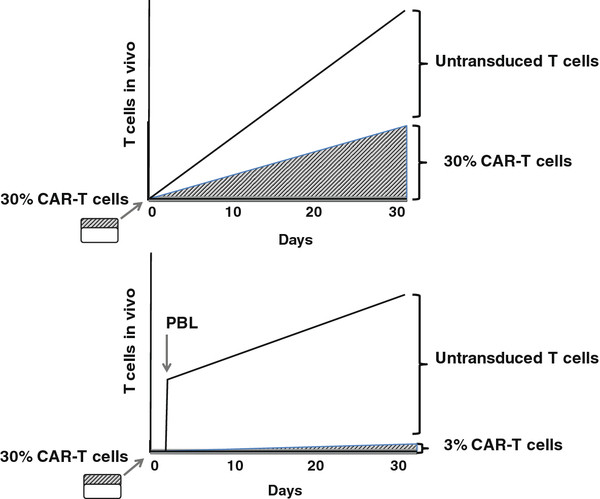
Modeling of CAR-T cell expansion in vivo in the lymphodepleted host without (above) and with (below) infusion of bulk untransduced PBL on Day 2.

To maintain a low frequency of CAR-T cells in the repopulated host, we introduced a design whereby after uncontrolled expansion of CAR-T cells is allowed for 48 hours, a bulk of untransduced autologous PBL is infused. Accordingly, subjects in the accelerated escalation phase (cohort 1) will receive one billion autologous untransduced PBL. To permit incremental expansion of CAR-T cells, subsequent cohorts will receive “decrescendo” untransduced PBL (1 billion, 700 million and none). The innovation lying behind the inclusion of untransduced PBL introduces a middle ground between completely lymphodepleting the host, whereby all space is made available for the proliferating CAR-T cells to occupy by expansion (leading to possible delayed toxicity), and infusing CAR-T cell in a non-depleted host, giving them no room to expand and thus likely limiting their therapeutic potential (Figure 4). If toxicity is still seen despite the above safety measures, CAR-T cells could also be controlled with corticosteroids and/or immunosuppressive drugs [44].

## Discussion

With compelling evidence that ovarian cancers are immunogenic tumors [45] and with the dramatic advances in laboratory and clinical procedures, new opportunities have been created for ovarian cancer immunotherapy [46,47]. In order to be effective, cancer immunotherapy is dependent on the presence of sufficient numbers of antitumor lymphocytes with appropriate homing and effector functions that enable them to seek out and destroy cancer cells in vivo. The adoptive transfer of ex vivo expanded, tumor-reactive T cells holds the potential of achieving this condition in a short period of time. However, adoptive T cell immunotherapeutic strategies utilizing naturally-occurring tumor-reactive T cells are limited by the availability of such T cells for administration and the downregulation of MHC class I molecules and antigen processing machinery by the tumor [48]. To obviate these obstacles CAR-T cells were designed to redirect the anti-tumor immune response to FRα expressed on the surface of ovarian cancer cells.

The objective of this study is to implement adoptive transfer of FRα CAR-T cells following transient lymphodepletion with cyclophosphamide/fludarabine and evaluate the safety and feasibility of this treatment for recurrent ovarian cancer patients. The ultimate goal of this approach is to induce tumor-specific immune response in patients with FRα-positive ovarian cancer and achieve a prolongation of progression-free survival in patients with a recurrent disease who otherwise do not respond to other therapies. We chose folate receptor as the target antigen because it is expressed at high levels in 90% of epithelial ovarian carcinoma [49], its expression is not significantly altered even after chemotherapy [50], and because it appears to be a safe target [33].

To successfully apply CAR-T cells in the clinic, transient lymphodepletion is necessary to create a favorable space and environment for the efficient expansion and activation of CAR-T cells. Lymphodepletion has many attributes such as reduction in the number of immunosuppressive regulatory T cells and enhancement of the half-life and effectiveness of adoptive T cell therapy [47]. Lymphodepletion also enhances transiently the availability of cytokines such as IL-7 and IL-15 [51]. The benefits of transient lymphodepletion prior to cell infusion are observed in an ongoing trial at the University of Pennsylvania where patients with myeloma who received autologous CD3/CD28-costimulated T cells 2 days after Busulfan showed improved engraftment compared to patients where T cells were given 12 days after chemotherapy [52,53].

We will also apply certain safety measures to mitigate the risks associated with this powerful therapy. We will start with a very low dose of CAR-T cells; we will administer higher doses in an incremental split-dose approach; and finally, we will infuse a large number of autologous PBLs two days after the CAR-T-infusion to suppress the potential overexpansion of CAR-T cells and to provide a safety mechanism.

We expect that FRα-specific CAR-T cells will persist in circulation for prolonged periods of time, just as it was seen in recent CD19 CAR trials. This will be evaluated using the same molecular methods, including flow cytometry and RT-PCR used previously [54]. However, it is possible that in the CD19 CAR trials, long persistence of CAR-T cells was allowed by the absence of HAMA due to the complete ablation of B cells, which ensued CD19 CAR-T cell therapy, as well as chronic antigen stimulation provided by ever emergent B cells arising from the bone marrow and attempting to reconstitute the host. In the proposed trial, the B cell compartment will be intact and HAMA [55] could develop and shorten the half-life of our FRα CAR-T cells. In that case, using a human scFv in the redesign of the CAR will be crucial to enable long-term engraftment of CAR-T cells.

We expect that the adoptively transferred CAR-T cells will traffic to the ovarian tumors deposits and will mediate regression of the cancer cells. Lymphocytes have complex trafficking and survival kinetics, but based on previous data from a trial with CAR-T cells at the University of Pennsylvania [22] we anticipate that the half-life of CAR-T cell following single infusion will be longer than that of normal CD8+ cells (half-life of normal CD8+ cells is 10 days) and that this therapy will provide a long lasting effect. Unlike antibody-mediated therapy, CAR-modified T cells have the potential to replicate and survive in vivo, and long-term presence could lead to sustained tumor control.

In summary, we hypothesize that the clinical efficacy of the proposed CAR-T cell regimen following transient lymphodepletion will be substantial and that the potential benefits outweigh the risks. CAR-T cells have been administered in patients with different types of tumors in different phase I trials. When patients with CLL were treated with CAR-T cells, the cell infusions had no acute toxic effects and the engineered cells persisted at high levels for 6 months in the blood and bone marrow and continued to express the CAR. If a lack of dose-limiting toxicities is confirmed in the present study, we will test whether exogenous cytokine support (e.g. IL-7) can enhance CAR-transduced T cell persistence and expand a CAR-transduced memory T cell population in vivo following adoptive transfer. A positive outcome from CAR-T therapy trials in terms of effective therapy, extension of progression free and overall survival in a recurrent setting would represent a major advancement for patients with advanced ovarian cancer.

## Abbreviations

CAR-T cell, Chimeric antigen receptor-expressing T cell; DLT, Dose limiting toxicity; ECOG, Eastern Cooperative Oncology Group; EOC, Epithelial ovarian cancer; EOS, End of study; FRα, Folate receptor-alpha; HAMA, Human anti-mouse antibody; irRC, Immune response related criteria; MHC, Major histocompatibility antigen; MTD, Moderate toxicity dose; NOAEL, No observable adverse effect level; OBD, Optimal biologic dose; PBL, Peripheral blood leukocytes; PET, Positron emission tomography; RECIST, Response evaluation criteria in solid tumors; scFv, Single chain variable fragment; TCR, T cell receptor; TIL, Tumor infiltrating lymphocytes; Treg, Regulatory T cell.

## Competing interests

The authors declare that they have no competing interests.
